# Modified Oral Health Assessment Tool (M-OHAT) for Residential Aged Care: A Co-Design Protocol

**DOI:** 10.3390/healthcare12191953

**Published:** 2024-09-30

**Authors:** Laura J. Ferris, Kristiana Ludlow, Nicole Walker, Andrew Georgiou, Julie D. Henry, Claudia Lopez Silva, Diep H. Ha, Nicole Stormon, Laurence J. Walsh, Saso Ivanovski, Christopher Sexton, Helena Silveira Schuch, Haitham Tuffaha, Angelique Zamora, Lyndal Pritchard, Loc G. Do

**Affiliations:** 1 School of Dentistry, The University of Queensland, Herston, QLD 4006, Australia; l.ferris@uq.edu.au (L.J.F.); n.walker4@uq.edu.au (N.W.); c.lopezsilva@uq.edu.au (C.L.S.); d.ha@uq.edu.au (D.H.H.); l.walsh@uq.edu.au (L.J.W.); s.ivanovski@uq.edu.au (S.I.); c.sexton@uq.edu.au (C.S.); h.schuch@uq.edu.au (H.S.S.); a.zamora@uq.edu.au (A.Z.); l.pritchard@uq.edu.au (L.P.); 2 School of Business, The University of Queensland, St. Lucia, QLD 4072, Australia; 3 School of Psychology, The University of Queensland, St. Lucia, QLD 4072, Australia; julie.henry@uq.edu.au; 4 Centre for Health Services Research, Faculty of Medicine, The University of Queensland, Woolloongabba, QLD 4102, Australia; k.ludlow@uq.edu.au; 5 Centre for Health Systems & Safety Research, Australian Institute of Health Innovation, Macquarie University, Macquarie Park, NSW 2109, Australia; andrew.georgiou@mq.edu.au; 6 Poche Centre for Indigenous Health, The University of Queensland, St. Lucia, QLD 4072, Australia; 7 Centre for the Business and Economics of Health, The University of Queensland, St. Lucia, QLD 4072, Australia; h.tuffaha@uq.edu.au; 8 School of Nursing, Midwifery and Social Work, The University of Queensland, St. Lucia, QLD 4072, Australia

**Keywords:** dental care, long-term care, non-dental staff, nursing homes, older adults, qualitative research

## Abstract

**Background**: Older adults in residential aged care facilities (RACFs) experience disproportionate levels of poor oral health relative to other groups in the general population, affecting their physical and mental wellbeing. The Oral Health Assessment Tool (OHAT) is a validated and widely used dental assessment tool; however, recent systematic reviews have identified shortcomings with respect to its measurement properties. **Objective**: The objective of this protocol is to provide a detailed overview of a multidisciplinary qualitative study that aims to (a) co-design and develop a modified OHAT for RACFs and (b) inform the development of an OHAT training package and implementation strategies. **Methods**: This study will utilize a co-design methodology with aged care residents, caregivers, staff members, and health professionals. The co-design workshops will: (1) investigate the barriers to and enablers of optimal oral healthcare in RACFs; and (2) co-design a modified version of the Oral Health Assessment Tool and a referral to treatment pathway that is appropriate for use in RACFs. The co-design workshops will facilitate group discussion and involve interactive activities using, for example, mind mapping and Sticky Notes. Qualitative data (transcripts and artefacts from co-design activities) will be analyzed in NVivo using an inductive codebook thematic analysis, specifically a template analysis. **Conclusion**: The findings of this study will inform a modified OHAT (M-OHAT), as well as future study phases regarding training and implementation strategies. It is expected that the M-OHAT will have enhanced usability and relevance to RACFs, facilitating the identification of poor oral health and timely referral to dental treatment.

## 1. Background

Poor oral health undermines broader physical and mental health. Oral health-related pain and infection can affect individuals’ ability to eat, speak, and engage socially with others. Oral health is a quality-of-life indicator and, therefore, the provision of oral healthcare should be considered a basic human right [1,2]. This protocol presents a brief literature review and methods for a study to co-design and develop a modified oral health assessment tool and referral to treatment pathway (e.g., dental services) for residential aged care facilities (RACFs), internationally known as nursing homes, care homes, and long-term care. Older adults living in RACFs have the highest prevalence of poor oral health in the general population, with dental caries (tooth decay) estimated to be as high as 75% [3,4]. RACFs are a unique setting for oral health assessment and treatment, given the diversity of residents’ health and wellbeing needs, care workforce characteristics, and the accessibility of oral health assessment and treatment [3,5,6,7,8,9,10,11]. Multiple factors contribute to disadvantaged oral health outcomes for older adults in RACFs relative to other community segments. More than two-thirds of people living in RACFs experience moderate to severe cognitive impairment [12], which can present significant challenges for residents to maintain their own oral self-care and to access and receive dental care [13,14,15]. High-quality longitudinal data have shown that a dementia diagnosis is associated with substantial subsequent declines in oral health utilization, with additional risk factors being rapid progressive cognitive impairment and frailty [16].

Social, organizational, and system factors also contribute to poor oral health in older adults living in RACFs [17]. Relevant social factors include norms about the importance or value of oral healthcare for older adults, along with generational and cultural differences in oral health literacy, access, past negative experiences, or historical dental-related trauma [18,19,20]. At an organizational level, staff members working in RACFs play a key role in facilitating oral healthcare and self-care. A Swedish study identified that RACF staff generally hold positive attitudes about the importance of oral healthcare for residents, but require additional support to enhance knowledge and training in implementation [11]. At a system level, aged care is a high-turnover sector. Staff often operate under strain amid the diverse health needs of aged care residents and systemic imperatives to attend to a range of care needs, limited by time and resource constraints [17,21,22,23]. Limited time and a lack of staff are two factors commonly reported to contribute to poor oral care in RACFs [21]. Facility location is another consideration in oral health resourcing and outcomes, with RACFs in regional and rural settings more likely to be impacted by low staffing, high turnover, and under-resourcing [24], alongside more sparse oral health services infrastructure [25]. These factors together create complex barriers to good oral health for older adults in RACFs, such that in many instances oral healthcare needs are left unmet or are unknown [21]. Good oral health is an opportunity to reduce the healthcare burden at the individual, organizational, and public health system levels [26]. Acceptable and effective oral health assessment is therefore key to addressing unmet needs. There have been several instruments developed for oral health assessment [27,28,29,30]; one such instrument is the Oral Health Assessment Tool (OHAT) [31]. The OHAT was designed to be used by those without dental training, including nursing assistants and registered nurses [31]. It has been implemented in a range of oral health contexts, including with older adults and in RACFs [32,33,34,35,36]. The OHAT collects information about an individual (e.g., behaviour, abilities, needs) and assesses oral health across eight domains: lips, tongue, gums and oral tissue, saliva, natural teeth, dentures, oral cleanliness, and dental pain. These domains are assessed against a traffic light system of green = healthy, yellow = changes from normal, and red = unhealthy, with the latter rating indicating that referral to a dental practitioner is needed. 

The OHAT was found to have acceptable measurement properties in its original assessment [31], and has been identified as one of the most complete oral health assessment tools for use by non-dental healthcare professionals with older adults [28]. However, after nearly 20 years since the publication of the original version [31], there is an opportunity to enhance and update the OHAT to better meet staff and resident needs in the residential aged care context and to keep pace with contemporary expectations for methodological quality in measurement [28,29]. For example, Rodrigues et al. (2023) identified that the OHAT has moderate-quality evidence showing insufficient internal consistency and sufficient evidence showing construct validity on oral health condition, but not dental pain. Moreover, when compared with other assessment tools, the OHAT does not measure items such as breath or chewing problems (although chewing is listed in the functional items along with grinding) [29]. Furthermore, the OHAT has the potential to be a very valuable tool for oral health screening in aged care, but barriers, enablers, and the overall suitability of this tool within residential aged care have yet to be systematically identified and assessed, particularly from consumer- and staff-informed perspectives. 

The objective of this protocol is to provide a detailed overview of a multidisciplinary qualitative study that aims to (a) co-design and develop a modified OHAT for RACFs and (b) inform the development of an OHAT training package and implementation strategies. The proposed study will utilise the OHAT [31], enriched with functional data and RACF staff and consumer perspectives to create a modified OHAT (M-OHAT). The study forms part of a larger multidisciplinary project to provide older adults living in rural, regional, and remote RACFs with access to effective and affordable dental care packages to arrest and seal untreated dental caries. The co-design phase will establish an industry- and consumer-informed foundation for oral health assessment and referral to treatment pathways, tailored for RACFs. 

## 2. Materials and Methods

### 2.1. Study Design

This qualitative study will utilise co-design principles across two stages: first, co-design workshops and semi-structured interviews with RACF consumers (residents, informal caregivers), RACF staff members, and health professionals (e.g., dentists, general practice doctors); and second, modification of the OHAT, with a view to informing the development of a RACF staff training package. This study is pre-registered on the Open Science Framework (https://osf.io/2kynu). 

Co-design involves actively collaborating with end-users and relevant stakeholders in research and design in meaningful ways [37]. In co-design, end-users are considered equal partners in design processes, facilitating a designing *with* rather than a designing *for* approach [38]. This research project will be guided by the *Beyond Sticky Notes* six co-design mindsets: (1) elevating lived experience, (2) practising curiosity, (3) offering generous hospitality, (4) being in the grey (being okay with uncertainty), (5) learning through doing, and (6) valuing many perspectives [39].

### 2.2. Participants

Participants will be eligible RACF staff members, health professionals, and consumers at participating RACFs in southern Queensland, Australia. Table 1 sets out the eligibility criteria for each participant type. Co-design workshops will be held separately for each participant type to reduce power imbalances and ensure that participants are comfortable speaking openly. One workshop will be held with health professionals, and at least two workshops will be held with RACF staff members and with consumers. It is anticipated that a minimum of 32 participants will be recruited, with 6–8 participants in each workshop. 

### 2.3. Materials and Data Collection

#### 2.3.1. Demographics

Demographic data will be collected in a brief survey prior to participation.

#### 2.3.2. Co-Design

Interactive workshops will utilise visual and verbal techniques to elicit barriers, facilitators, perspectives, and experiences relevant to oral health in RACFs, and specifically seek perspectives on the OHAT, including design ideas for modifications. Separate groups will be convened for consumers (residents and informal caregivers) and RACF staff members. Semi-structured interviews will be offered for any participant not wishing to engage in a group format. Workshops and interviews will be video- and audio- recorded. During the workshops, the research team will present the purpose of the study and the features of the OHAT, followed by a needs assessment, interactive design activities (e.g., mind mapping, use of Sticky Notes), and discussions of dental care needs and experiences with barriers to and facilitators of oral health and oral health assessment in RACFs. Workshops with RACF staff members and health professionals will additionally focus on the development of implementation strategies that can increase the uptake of oral health assessments, as well as staff members’ opinions on the training approach and resources for the M-OHAT. Workshop facilitators will write reflective field notes on group dynamics and their initial impressions of emerging patterns in the data.

The co-design phase aims to elicit sector-specific challenges that may impede routine use of the OHAT in RACFs. These could include resources, work environment, staff factors, or resident factors [21]. Perspectives, experiences, and design ideas will inform modifications to the tool to facilitate broader implementation in RACFs. 

#### 2.3.3. Tool Modification and Training Development

Co-design insights will be integrated with clinical and functional data to implement modifications to the OHAT to create the M-OHAT. Referral pathways will be articulated based on an integration of evidence-based practice, team expertise, and co-design learnings, which will provide process and content guidance on oral healthcare options once a resident has been assessed by RACF staff using the M-OHAT. In future stages of the project, an M-OHAT training package will be co-designed and developed with researchers, psychologists, and oral health professionals within the research team, in collaboration with RACF staff members.

### 2.4. Sample Size

Co-design samples sizes are designed to achieve thematic saturation and adhere to disciplinary conventions [40,41,42]. Based on previous research and our teams’ experience, we will aim for a total of *n* = 32 participants across five workshops, with the potential for 1:1 interviews [43,44,45]. 

### 2.5. Analysis

Co-design data from workshops and interviews (transcripts, audio/video recordings, mind maps, notes, and other artefacts) will be analysed using inductive template analysis, which is a type of codebook thematic analysis [40,42]. Member checking will be provided as an option for participants and communicated through the consent process, with participants afforded the opportunity to review or edit their responses prior to thematic analysis of the data. Next, three researchers with qualitative research experience will conduct the analysis. The researchers will familiarise themselves with the data by reviewing transcripts and artefacts. They will then independently code the data using an inductive approach. The researchers will meet to discuss their coding; identify and refine themes by grouping codes with similar meaning and reviewing reflective field notes; and collaboratively develop a coding template. This template will then be reapplied to all transcripts. Exploratory ancillary analyses may include post-hoc examination of video footage, for example, to ascertain observer ratings of apparent resident comfort during the workshops or observable resident-staff rapport.

## 3. Ethics and Dissemination

### 3.1. Ethical Considerations

This research has been approved by the relevant Human Research Ethics Committees (The University of Queensland, 2023/HE000093 on 18 May 2023; Queensland Health Darling Downs Hospital and Health Service HREC/2022/QTDD/94702 on 16 October 2023). Principal ethical considerations relate to participant vulnerability factors, including that participants may be (i) people with a cognitive impairment and/or mental illness, and (ii) people in dependent or unequal relationships [46]. The project aims to adhere to the principles of inclusion, support, and respect for participants falling within these categories. 

Study eligibility for residents includes cognitive screening to establish capacity to give informed consent to participate [47,48,49]. Participants may demonstrate their capacity to give informed consent, yet have some degree of cognitive impairment or experience mental illness [48,50,51]. Alternatively, they may be participating with the consent provided by their nominated EPOA or AHD. In the co-design process, participants with a cognitive impairment or mental illness will be supported to provide their personal experiences and opinions to the broader group, but will also be offered the opportunity to work with smaller groups (to overcome any larger group hesitation) or to have an individual interview instead, should this be their preference. Participants will be supported by trained research team members to answer surveys and will have the option to complete the survey items in a manner that suits their needs and abilities (e.g., verbally responding, having the survey read to them, responding electronically, responding with pen and paper) [52]. Participants will be assured that they may withdraw from the study at any time without the need to provide a reason. Wellness breaks will be scheduled or enacted whenever needed. While the workshop content is not expected to produce any discomfort beyond that of everyday living, it is important to acknowledge that some people may be uncomfortable or become upset in relation to incidental topics, such as discussing past negative dental health experiences [53,54]. These topics are not the focus of the research, but distress procedures will be in place to support any participant in need [55]. Trained mental health facilitators will be present at all workshops, and any person requiring additional supports will be referred to specified services. 

Aged care staff may fall into the category of people in dependent or unequal relationships [46]. Staff are employees of the participating RACFs and may inadvertently perceive that participation is required or expected. Staff participants will be assured that there is no obligation to take part, their decision to participate (or not) will not be made known to their employer, their data will remain anonymous, participation will not affect their relationship with any organization, and they may withdraw from the study at any time without prejudice. 

Consumer participants may also be people in dependent or unequal relationships [46]. Consumers are residents or the informal caregivers of residents receiving care and services from the participating RACFs and they may inadvertently perceive that participation is required or expected. Consumer participants will be assured that there is no obligation to take part, their decision to participate (or not) will remain private, their data will remain anonymous, participation will not affect their relationship with any organization, and they may withdraw from the study at any time without prejudice. Participants will be remunerated at standard consumer participant rates in acknowledgement of their time [56]; for staff, remuneration will be undertaken to the extent permitted by organizational policies. 

### 3.2. Data Curation and Dissemination

Researchers will collect and de-identify data for analysis. Project data will be stored in The University of Queensland’s secure server, Research Data Management, in line with the Australian Code for the Responsible Conduct of Research. It is anticipated that the research findings will be published (e.g., in peer-reviewed journals and newsletters) and presented at public forums (e.g., conferences, seminars, webinars and workshops). Aggregate (non-identifying) findings will be reported to RACFs and made available to any participant who indicates their interest. In any publication or presentation, information will be reported in such a way that participants cannot be identified, except with their express permission or request.

## 4. Discussion

### 4.1. Significance and Expected Outcomes

The co-design workshops will elicit participants’ perspectives and experiences regarding the barriers to and facilitators of oral healthcare in RACFs, including the use of the OHAT in its current form. The workshops will also generate design ideas for modifications of the OHAT. These design ideas will be discussed amongst the research team, including members with dental expertise, which will inform iterative rounds of modifications. The M-OHAT will then be presented back to the aged care partners at Scientific Advisory Committee meetings for feedback. The research findings will be used to create the M-OHAT and inform future study phases regarding the training package, implementation strategies to support the adoption and uptake of the M-OHAT, and referral pathways into routine practice in RACFs. The M-OHAT will be piloted in RACFs as part of the larger research project. It is anticipated that the modifications derived from this study will enhance the usability and relevance of the OHAT in RACFs. It is expected that this co-design study will establish an industry- and consumer-informed foundation for oral health assessment tailored for residential aged care settings, and will facilitate identification of poor oral health and timely referral to dental treatment.

### 4.2. Strengths and Limitations of This Study

This pre-registered study is the first to embed co-design principles into the modification of an oral health assessment tool. A strength of this project is the use of a co-design methodology with end-users including residential aged care consumers (residents and caregivers) and frontline staff. The integration of lived experience, alongside interdisciplinary perspectives from oral health and dentistry, nursing, aged care, psychology, and public health, is anticipated to enhance the relevance of the modified assessment tool to RACFs and facilitate usability. While this study will recruit a range of stakeholders, it is limited to participating RACFs in Queensland, Australia. Therefore, the breadth of RACFs (e.g., RACFs in other States and Territories) will not be captured. It is not anticipated that perspectives and design ideas for the modified OHAT will greatly differ between facilities/location, however, it is likely that implementation and training needs will. This study will inform future research phases of the larger research project that address training with, and the implementation and scaling-up of, the M-OHAT. 

## 5. Conclusions

Oral health is a priority for the health and wellbeing of older adults living in RACFs. The findings of the study presented in this protocol will directly support the modification of an oral health assessment tool (M-OHAT) for RACFs and inform future study phases to develop RACF staff training and implementation strategies. Altogether, the research is expected to enhance the identification of poor oral health and facilitate timely referral to dental treatment for people living in RACFs.

## Figures and Tables

**Table 1:** Eligibility criteria for consumers (residents, informal caregivers), RACF staff members and health professionals.

Participant Type	Inclusion Criteria	Exclusion Criteria
Consumers (residents)	Aged 65 years or older, or 55 years or older if identifying as Aboriginal and/or Torres Strait Islander.	Aged under 65 years old, or under 54 years old if identifying as Aboriginal and/or Torres Strait Islander.
	Resides permanently in a participating RACF.	Does not reside in a participating RACF, e.g., respite care, community, home care and hospitals; or resides in a RACF not participating in the research project.
	Able and willing to provide informed consent. This will be determined via the modified Telephone Interview for Cognitive Status (TICS-M, Australian Version), with a cut off score of 21 or greater supporting presence of capacity to give informed consent. For those who do not meet the eligibility criteria in cognitive screening and for whom an Enduring Power of Attorney (EPOA) and/or Advance Health Directive (AHD) is in place, consent may be sought from the EPOA/AHD.	Unable or unwilling to provide informed consent, including those with scores <21 on the TICS-M, Australian Version; or does not have a EPOA or AHD; or EPOA or AHD does not consent to the study.
	Able and willing to participate in a workshop or an informal interview.	Unable or not willing to participate in a workshop or an informal interview.
Consumers (informal caregivers)	Aged 18 years or older.	Aged under 18 years old.
	Provides informal care to a permanent resident in a participating RACF.	Does not provide informal care to a permanent resident in a participating RACF.
	Able and willing to provide informed consent.	Unable or not willing to provide informed consent.
Age care staff and other health professionals	Employed by a participating RACF or associated care team (e.g., GP, dentist).	Does not work in a RACF or associated care team.
	Has permission from the aged care provider to participate in the study, or willing to participate in non-work time where necessary.	Does not have permission from the aged care provider to participate in the study or is not willing to participate in non-work time where necessary.
	Able and willing to provide informed consent.	Unable or not willing to provide informed consent.

Abbreviations: AHD = Advance Health Directive; EPOA = Enduring Power of Attorney; GP = General Practitioner; RACF = Residential aged care facility; TICS-M = Telephone Interview for Cognitive Status [modified version].

## Data Availability

This protocol does not contain data.
